# Development of Deep Learning Models for AI-Enhanced Telemedicine in Nursing Home Care

**DOI:** 10.3390/jcm15020828

**Published:** 2026-01-20

**Authors:** Nuria Luque-Reigal, Vanesa Cantón-Habas, Manuel Rich-Ruiz, Ginés Sabater-García, Álvaro Cosculluela-Fernández, José Luis Ávila-Jiménez

**Affiliations:** 1 Department of Nursing, Pharmacology and Physiotherapy, Faculty of Medicine and Nursing, University of Cordoba, 14071 Córdoba, Spain; n62luren@uco.es (N.L.-R.); en1rirum@uco.es (M.R.-R.); 2 Instituto Maimónides de Investigación Biomédica de Córdoba (IMIBIC), 14004 Córdoba, Spain; 3 Hospital Universitario Reina Sofía, 14004 Córdoba, Spain; 4 CIBER on Fragility and Healthy Aging (CIBERFES), 28029 Madrid, Spain; 5 Grupo Vitalia, 50001 Zaragoza, Spain; gsabater@vitaliaplus.es (G.S.-G.); acosculluela@vitaliaplus.es (Á.C.-F.); 6 Department of Electronic and Computer Engineering, Andalusian Research Institute in Data Science and Computational Intelligence (DaSCI), University of Cordoba, 14071 Córdoba, Spain; jlavila@uco.es

**Keywords:** nursing homes, telemedicine, deep learning, acute health events, aged

## Abstract

**Background/Objectives:** Acute health events in institutionalized older adults often lead to avoidable hospital referrals, requiring rapid, accurate remote decision-making. Telemedicine has become a key tool to improve assessment and care continuity in nursing homes. This study aimed to evaluate outcomes associated with telemedicine-supported management of acute events in residential care facilities for older adults and to develop a deep learning model to classify episodes and predict hospital referrals. **Methods:** A quasi-experimental study analyzed 5202 acute events managed via a 24/7 telemedicine system in Vitalia nursing homes (January–October 2024). The dataset included demographics, comorbidities, vital signs, event characteristics, and outcomes. Data preprocessing involved imputation, normalization, encoding, and dimensionality reduction via Truncated SVD (200 components). Given the imbalance in referral outcomes (~10%), several resampling techniques (SMOTE, SMOTEENN, SMOTETomek) were applied. A deep feedforward neural network (256–128–64 units with Batch Normalization, LeakyReLU, Dropout, AdamW) was trained using stratified splits (70/10/20) and optimized via cross-validation. **Results:** Telemedicine enabled the resolution of approximately 90% of acute events within the residential setting, reducing reliance on emergency services. The deep learning model outperformed traditional algorithms, achieving its best performance with SMOTEENN preprocessing (AUC = 0.91, accuracy = 0.88). The proposed model achieved higher overall performance than baseline classifiers, providing a more balanced precision–specificity trade-off for hospital referral prediction, with an F1-score of 0.63. **Conclusions:** Telemedicine-enabled acute care, strengthened by a robust deep learning classifier, offers a reliable strategy to enhance triage accuracy, reduce unnecessary transfers, and optimize clinical decision-making in nursing homes. These findings support the integration of AI-assisted telemedicine systems into long-term care workflows.

## 1. Introduction

The contemporary pace of life, characterized by extended working hours and school activities that occupy a significant portion of family time, has considerably reduced the time people spend at home, making work–life balance increasingly difficult [1]. This situation has led to a greater dependence on external resources for the care of children, older adults, and household tasks, not only in domestic chores but also in direct and specialized care. Traditionally, care for older adults has been provided within the family home, mainly by women [2,3], who in many cases interrupted their professional activities to assume exclusive responsibility for dependent family members [4].

Economic and health advances have contributed to a significant increase in life expectancy [5]. However, this increase does not always translate into years lived in full health, resulting in higher levels of dependency [6] and the need for specialized care. In this context, formal caregivers, defined as professionals without direct kinship who have specific training and provide care in exchange for compensation, have become a fundamental resource to complement family care [7]. Nevertheless, family homes often lack the necessary infrastructure and resources to comprehensively care for older adults with complex needs [8,9], frequently leading to consideration of institutionalization in residential care facilities as a viable alternative to ensure quality of life and safety.

Residential care facilities for older adults offer comprehensive and continuous care, with either permanent or temporary stays, and provide multiple services aimed at preserving autonomy and improving residents’ quality of life [10]. According to data from the National Statistics Institute (INE), in Spain, there were 5188 residential care centers in 2022, with a capacity for 381,514 older adults, reflecting sustained growth in both the number of centers and available places [11]. This increase partly responds to demographic changes and population aging, as well as the high prevalence of chronic diseases and multimorbidity among the resident population [12].

The transformation of the epidemiological structure in residential care facilities has altered the user profile. While residents with moderate dependency previously predominated, the majority now present complex needs that include high functional and cognitive dependency, polypharmacy, and multiple comorbidities [13]. This profile increases vulnerability to acute health events such as falls, respiratory infections, metabolic crises, or cardiovascular decompensations, which can lead to hospitalizations, medical complications, and functional decline [14]. Therefore, care for this population requires continuous and specialized attention, posing a significant challenge for residential centers where healthcare resources are limited and staff distribution exhibits considerable inequalities [15].

In most centers, nursing assistant technicians represent the largest and continuously available workforce [16]. Nursing staff are primarily concentrated during morning and afternoon shifts, while medical personnel have limited presence, generally only during morning hours [17]. This labor organization contributes to what has been termed the “care crisis [18], characterized by a shortage of qualified personnel, high workload, and elevated turnover rates [19,20], factors that hinder the provision of individualized care and rapid response to acute events, increasing the likelihood of avoidable hospital referrals [16].

In response to these challenges, telemedicine emerges as a strategic tool to strengthen clinical response capacity in residential care facilities. Defined as the use of information and communication technologies (ICTs) for the provision of remote health services [21], telemedicine enables rapid and coordinated evaluation of acute events, continuous monitoring of vital signs, and multidisciplinary communication among nursing staff, primary care physicians, and hospital specialists [22,23,24]. This technology contributes to improving safety, efficiency, and continuity of care, allowing for more person-centered attention [24,25].

The integration of Artificial Intelligence (AI) models into telemedicine platforms enhances their utility by enabling predictive analytics and support for clinical decision-making [26]. These models require large volumes of historical and real-time clinical data for training, and their application facilitates the standardization of care protocols, reduction in clinical variability, and optimization of healthcare resource allocation [27]. Thus, AI models enable a proactive and predictive approach to managing acute events, helping to reduce avoidable hospitalizations, improve resident safety, and promote a more efficient and person-centered care model [28].

In this context, the combination of telemedicine and AI tools represents a significant opportunity to transform care in residential facilities, providing a faster and more coordinated response to the complex needs of the dependent elderly population [29]. The implementation of these innovative approaches is key to addressing the challenges posed by population aging, multimorbidity, and the increasing demand for specialized care, ensuring comprehensive, safe, and efficient care [30].

Previous studies on telemedicine in nursing homes have mainly focused on feasibility, clinical outcomes, user acceptance, and reductions in hospital transfers, often through descriptive analyses or protocol-driven interventions rather than predictive decision support systems [22,23,24,25,31,32,33,34,35]. These works consistently report the potential of telemedicine to improve access to care and reduce unnecessary hospitalizations, but they typically do not address real-time risk stratification or referral prediction during acute events in residential care settings.

In parallel, machine learning and deep learning approaches have been extensively explored in healthcare for disease prediction, clinical decision support, and outcome forecasting [26,27,28,29,30,36,37,38]. However, most of these studies rely on electronic health records, imaging data, or population-level datasets, with limited integration into telemedicine workflows or nursing home contexts. Moreover, issues related to severe class imbalance and realistic evaluation under rare-event conditions are often underexplored.

In contrast to these prior approaches, the present study integrates a large-scale, real-world telemedicine dataset collected in nursing homes with advanced machine learning models explicitly designed to support hospital referral assessment during acute events. Beyond reporting global performance metrics, this work systematically compares multiple rebalancing strategies and model architectures under pronounced class imbalance, while explicitly analyzing clinically relevant trade-offs between sensitivity, precision, and false alarm reduction. This positioning highlights the novelty of the proposed framework in bridging telemedicine-enabled acute care with robust machine learning evaluation in a real-world institutional setting.

The objective of the study was therefore: (1) to evaluate outcomes associated with telemedicine-supported management of acute events in residential care facilities for older adults; and (2) to propose a deep learning model for the classification of acute health events and to evaluate its potential impact on clinical decision support.

## 2. Materials and Methods

### 2.1. Dataset Description and Preprocessing

A single-arm, real-world, quasi-experimental, retrospective implementation study was conducted, focused on the analysis of outcomes observed during telemedicine-supported management of acute events between January and October 2024. The study population consisted of institutionalized older adults aged 65 years and above residing in Vitalia company’s residential care centers, who experienced acute health episodes requiring medical assistance delivered through telemedicine. During the study period, Vitalia residential care centers accommodated 9687 residents, of whom those experiencing acute health events managed via telemedicine constituted the study sample.

A total of 5202 records were collected, encompassing a wide range of variables extracted from patients’ clinical records. These variables included sociodemographic data such as age, sex, and location of the residential facility. Clinical variables primarily comprised coded according to ICD-10 and pharmacological treatment systematically recorded by active pharmaceutical ingredient.

Baseline physiological parameters were also recorded, including oxygen saturation, systolic and diastolic blood pressure, heart rate, blood glucose levels, height, weight, and body temperature.

In addition to clinical data, information specific to the acute event was gathered: reason for consultation, exact date and time of occurrence, professional category and role of the person identifying the acute event, and the final resolution of the event.

For acute events, the dataset includes information on the primary reason for each consultation, organized into predefined clinical categories (e.g., infections, trauma, and metabolic conditions), along with recorded signs and symptoms, the diagnostic tools employed, and the duration of each intervention. In addition, variables related to clinical processes and outcomes capture details about the attending physician, such as professional background and experience, as well as the actions undertaken during the telemedicine encounter, thereby supporting an integrated analysis of care workflows and clinical decision-making.

Telemedicine was used both during periods when on-site physicians were unavailable (e.g., evenings, nights, weekends) and as a complementary tool during regular working hours to support on-site clinical decision-making.

The resolution was classified into two categories: (1) requirement for assistance by out-of-hospital emergency services at the residential facility without the need for hospital transfer, and (2) necessity of referral to a hospital. Moreover, whether the episode necessitated a modification of the treatment regimen within the residential center was documented.

All variables were collected from both clinical records and the data registry provided by Comitas e-Health mobile cart following telemedicine use. Data were captured at the time of the acute event and at a follow-up interval of 24 to 72 h in cases where hospital referral occurred. In addition, for patients whose acute events were resolved within the residential facility, follow-up consisted of routine clinical monitoring by nursing staff, with reassessment through telemedicine if symptom persistence or clinical deterioration occurred. Referrals were further categorized as necessary referrals if the hospital visit involved diagnostic tests or treatments unavailable within the residential care setting through telemedicine services.

The intervention relied on continuous telemedicine support delivered via a Comitas e-Health mobile cart (https://comitas.es/carro-telemedicina, accessed on 5 December 2025). The system consists of a self-contained, mobile workstation with adjustable height that integrates a range of clinical diagnostic tools, including imaging, cardiopulmonary monitoring, and vital-sign assessment devices, to enable remote clinical evaluation. Audiovisual communication with off-site specialists is supported through an integrated camera system and wireless connectivity, allowing synchronous consultations to be conducted reliably. Owing to its autonomous and ergonomic design, the platform can be rapidly deployed within nursing home settings, facilitating on-site specialist input and remote diagnostic assessment without requiring patient relocation.

**Figure 1:**
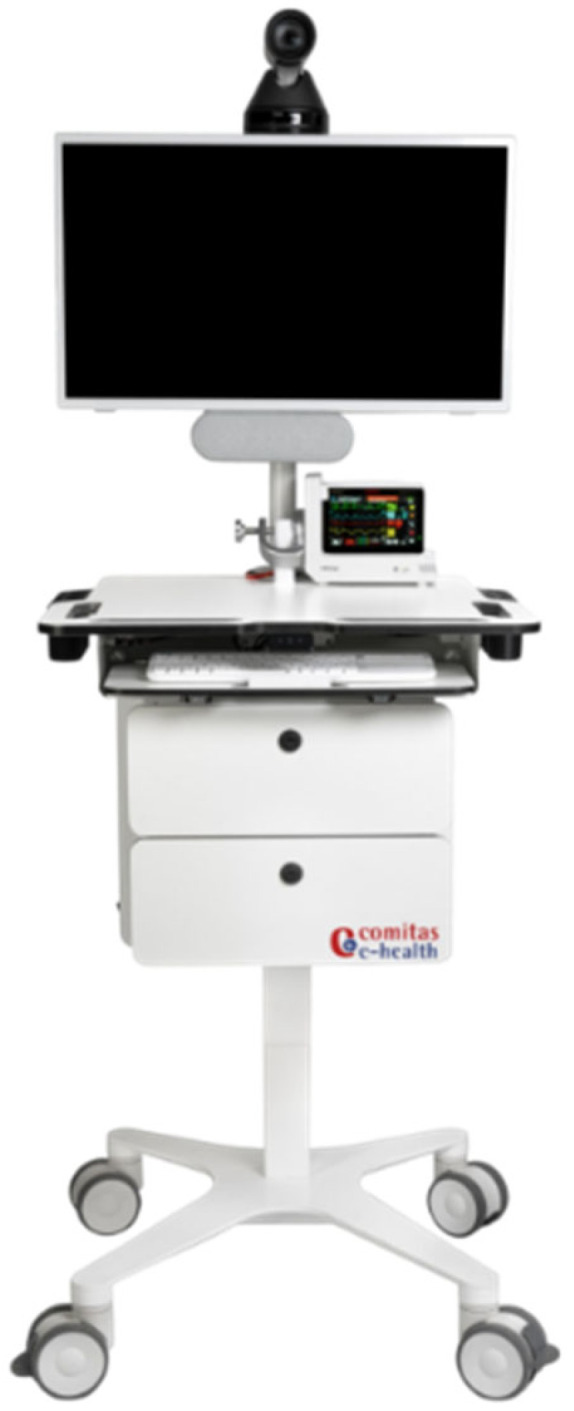
This is a figure showing the Comitas e-Health mobile telemedicine cart.

Prior to implementation, comprehensive training was provided to clinical staff (including nursing staff, nursing assistants, and on-site physicians) covering device operation and handling, data acquisition procedures, and integration of the telemedicine platform into routine clinical workflows, in order to ensure effective and efficient use (Figure 1).

Missing data were addressed using a structured preprocessing strategy. Numerical variables were completed by imputing the median value computed from the training subset, whereas categorical attributes were handled by assigning a dedicated category to represent missing entries before encoding.

All continuous inputs, including physiological measurements, were subsequently standardized using z-score scaling to facilitate model training. In addition, comorbid conditions originally coded under ICD-10 or SNOMED taxonomies were aggregated into higher-level clinical groups (such as cardiovascular, respiratory, metabolic, or infectious) in order to limit feature dimensionality and improve interpretability. Categorical variables were finally transformed into binary features through one-hot encoding.

To reduce dimensionality after one-hot encoding and to ensure computational efficiency, Truncated Singular Value Decomposition (SVD) was applied [39]. The number of retained components was set to 200, corresponding to approximately 99.9% of the cumulative explained variance. This choice was guided by the need to preserve nearly all the information contained in the original feature space while enabling stable and efficient training of machine learning models on a large, sparse dataset. Preliminary experiments with a lower number of components showed a noticeable degradation in predictive performance, whereas higher values did not yield additional gains, motivating the selected configuration.

The target variable corresponded to the resolution of each acute episode and was defined according to whether hospital referral was required or not. The resulting class distribution was highly imbalanced, with most cases being resolved on site (approximately 90%) and only a minority leading to referral (around 10%). To address this imbalance, several resampling strategies were applied, including Synthetic Minority Over-sampling Technique (SMOTE) [40], SMOTEENN33 [41], and SMOTETomek [41]. These methods either generate additional samples for the minority class or remove ambiguous majority-class instances, thereby improving sensitivity to referral cases and reducing bias during model training.

To avoid any potential data leakage, all resampling techniques were applied exclusively to the training data after dataset splitting. The dataset was first partitioned into training, validation, and test sets using stratified sampling, and neither the validation nor the independent test sets were subjected to any resampling. During cross-validation, resampling was performed independently within each training fold, ensuring that synthetic samples were generated only from the corresponding fold-specific training data.

### 2.2. Neural Network Design

The model developed in this work is a deep feedforward neural network based on a multilayer perceptron architecture with three hidden layers consisting of 256, 128, and 64 neurons. To improve training dynamics and enhance numerical stability, Batch Normalization [42] is applied after each hidden layer, helping to mitigate internal covariate shift. Nonlinear modeling capacity is provided through the use of LeakyReLU activation functions [43], which facilitate gradient flow and reduce the risk of vanishing gradients in deeper networks. To improve generalization and limit overfitting—particularly in the presence of high-dimensional inputs and class imbalance—dropout layers are included following each fully connected layer. Model optimization is performed using the AdamW algorithm [44], which separates weight decay from gradient-based parameter updates, leading to more stable and efficient convergence in large feature spaces.

This model configuration was chosen because, when combined with dimensionality reduction techniques and resampling strategies to address class imbalance, it proved effective at identifying subtle and clinically relevant patterns within the telemedicine data. A systematic hyperparameter optimization process was conducted, including the adjustment of learning rate, batch size, and dropout probabilities through cross-validation. Several alternative network designs were evaluated, varying both the depth of the architecture (from two to four hidden layers) and the number of neurons per layer (ranging from 64 to 512), as well as exploring different activation functions and regularization settings. Across these experiments, the final selected model consistently achieved the most favorable trade-off between ROC AUC, F1-score, and generalization performance on the validation set, outperforming both simpler configurations and more complex variants. A summary of the final architecture, training strategy, and regularization parameters is provided in Table 1.

The available data were divided into separate training, validation, and test sets, comprising 70%, 10%, and 20% of the samples, respectively. A stratified splitting strategy was applied to ensure that the relative frequency of the minority class (hospital referrals) was consistently maintained across all subsets, while the independent test set was used exclusively for final performance evaluation to ensure an unbiased assessment of real-world applicability. This methodological workflow supports reliable benchmarking and strengthens the reproducibility of results.

### 2.3. Baselines

To establish a robust comparison framework, we assessed a range of classification methods to estimate the likelihood of hospital referral in institutionalized patients presenting with acute medical conditions. Logistic Regression was included as a baseline model due to its interpretability and simplicity, providing a transparent and clinically understandable reference point against which more complex methods could be benchmarked [45]. In addition, a Random Forest model was included, using an ensemble of decision trees to improve predictive performance, capture non-linear feature interactions, and mitigate overfitting through bootstrap aggregation [46]. To further address non-linear decision boundaries, a Support Vector Machine with a Radial Basis Function (RBF) kernel was incorporated, enabling the projection of input features into higher-dimensional spaces where optimal margins can be identified [47]. Finally, the K-Nearest Neighbors classifier was evaluated as a non-parametric, instance-based learning approach that assigns class labels based on distance metrics relative to neighboring training examples, providing a useful contrast in terms of computational behavior and decision structure [48].

Parameter tuning was performed for all baseline models to ensure a fair comparison with the proposed neural network. Regularization strengths and class weights were optimized for Logistic Regression, while the number of trees, maximum depth, and minimum samples per split were adjusted through grid search for the Random Forest. For the SVM model, the penalty parameter C and kernel coefficient\gamma were explored across multiple scales, and for the KNN classifier, an optimal value for k and distance metric selection were determined using cross-validation. This systematic optimization aimed to balance performance and computational cost while obtaining representative results for each algorithm. Together, these baselines provide a comprehensive reference spectrum ranging from interpretable linear models to more flexible non-parametric approaches, enabling a rigorous assessment of the incremental value provided by the deep-learning architecture.

### 2.4. Performance Metrics

In this study, we employed a set of widely accepted classification metrics to systematically evaluate model performance. Specifically, accuracy, precision, and recall, derived from the confusion matrix, were used in conjunction with the Area Under the Receiver Operating Characteristic Curve (AUC). Accuracy, defined as the proportion of correctly classified instances among all predictions, provides a global estimate of the model’s overall predictive performance. However, because accuracy alone can be misleading in the presence of class imbalance, as is the case in our dataset, where the distribution of classes is substantially imbalanced, additional metrics were incorporated.

$$Accuracy=\frac{TP+TN}{TP+TN+FP+FN}$$

Precision quantifies the proportion of correctly identified positive cases relative to all instances predicted as positive, thereby reflecting the model’s ability to minimize false positive outcomes. This is particularly relevant in clinical contexts where incorrect positive classifications may lead to unnecessary diagnostic procedures or interventions.

$$Precision=\frac{TP}{TP+FP}$$

Recall measures the proportion of true positive cases that are correctly identified by the model. A high recall is essential in settings where failing to detect positive cases could have significant clinical consequences, such as missing early indicators of disease or failing to identify at-risk patients.

$$Recall=\frac{TP}{TP+FN}$$

In addition to these discrete metrics, the AUC was used to assess the model’s discriminatory capacity across a range of decision thresholds. By integrating the true positive rate and the false positive rate over the entire operating range, the AUC provides a threshold-independent evaluation of performance, which is particularly valuable in medical applications where the optimal decision threshold may vary according to clinical priorities or risk-benefit considerations.

$$AUC=\int_{0}^{1}TPR\;\left(FPR\right)\;d(FPR)$$

Collectively, these metrics offer a robust and comprehensive evaluation framework, ensuring that both the predictive reliability and clinical relevance of the models are adequately captured.

### 2.5. Ethical Aspects

Regarding ethical aspects, the study adhered to the principles of Good Clinical Practice and complied at all times with the ethical guidelines outlined in the Belmont Report and the Declaration of Helsinki, including their latest updates, as well as the Oviedo Convention. Data confidentiality was strictly maintained through anonymization in the database, in accordance with Royal Decree 1720/2007, which implements Organic Law 3/2018 of December 5 on the Protection of Personal Data and Guarantee of Digital Rights, Law 14/2007 of July 3 on Biomedical Research, and Law 41/2002 of November 14 regulating Patient Autonomy. The study was submitted to the Ethics Committee of Andalusia, which issued a favorable opinion under the communication code: SICEIA-2024-002742.

### 2.6. Implementation

The predictive model was implemented using a reproducible and modular pipeline designed to integrate seamlessly with the telemedicine infrastructure deployed in the participating nursing homes. All data preprocessing, feature engineering, dimensionality reduction, and model training procedures were developed in Python (version 3.x), using open-source scientific libraries including pandas, NumPy, scikit-learn, and TensorFlow/Keras.

All preprocessing steps, including scaling, dimensionality reduction, and resampling, were implemented within a unified pipeline to ensure that transformations were fitted exclusively on training data, thereby preventing information leakage across data splits.

The implementation workflow was executed in four main stages. First, raw telemedicine records were automatically ingested from the telemedicine platform and processed through a data-cleaning module that handled missing values, harmonized variable, and applied standardized encoding and normalization procedures. Dimensionality reduction was performed to optimize computational efficiency and mitigate the high sparsity resulting from one-hot encoding of categorical variables.

**Figure 2:**
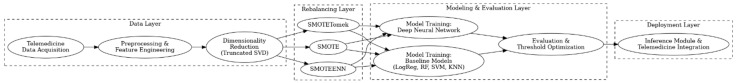
Experimental pipeline.

In order to avoid bias toward the majority class and enhance minority-class sensitivity, several resampling techniques were incorporated into the pipeline, including SMOTE, SMOTEENN, and SMOTETomek to generate synthetic minority examples and removed borderline or noisy majority instances, thereby improving class separability before model training (Figure 2).

Second, the modeling block consisted of a deep feedforward neural network trained with class-balanced weighting and validated through stratified partitioning to preserve the minority positive class. Training was performed on a workstation equipped with an NVIDIA GTX-class GPU.

Third, the inference module was implemented as a lightweight Python service capable of loading the trained model and associated preprocessing objects (SVD transformer, scaler, and feature encoder). The complete pipeline was designed to allow future clinical integration through RESTful APIs.

## 3. Results

### 3.1. Epidemiological Characteristics and Clinical Outcomes of Acute Events Managed via Telemedicine

A total of 5202 acute health events were managed via telemedicine during the study period. Female patients accounted for 70.60% (n = 3671) of these cases, while male patients represented 29.40% (n = 1529).

**Figure 3:**
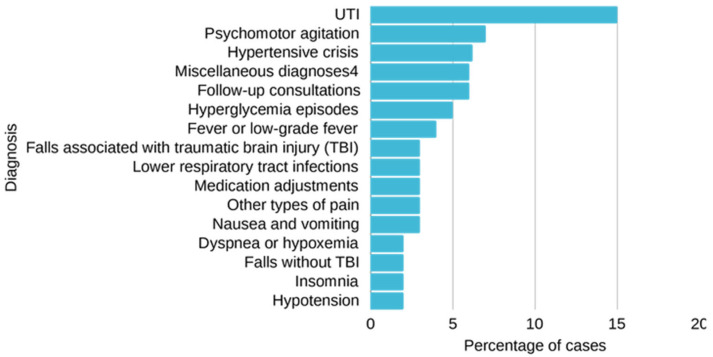
Most frequent acute health conditions.

The ten most prevalent acute health conditions, ranked by frequency, were urinary tract infection (UTI), psychomotor agitation, hypertensive crisis, miscellaneous diagnoses, follow-up consultations, hyperglycemia episodes, fever or low-grade fever, falls associated with traumatic brain injury (TBI), lower respiratory tract infections, medication adjustments, other types of pain, nausea and vomiting, dyspnea or hypoxemia, falls without TBI, insomnia, and hypotension (Figure 3).

Regarding clinical outcomes, treatment protocols that facilitated the management of patients within the residential facility were implemented in 78.89% of cases (n = 4097). Emergency interventions were necessary in a minority of cases, accounting for 2.18% (n = 113), with all emergency services interventions conducted on-site, resulting in resolution within the residential care facility without the need for hospital transfer. Hospital referrals were documented in 7.40% of cases (n = 384). These outcomes correspond to different stages of the clinical course and were recorded independently; therefore, individual cases could be represented in more than one outcome category.

### 3.2. Models’ Results

The results summarized in Table 2 reveal marked differences in predictive performance across the evaluated classification approaches for hospital referral. Within the group of conventional machine learning models, the Random Forest algorithm achieved the strongest performance, reaching the highest F1-score (0.3148), ROC AUC (0.7784), and overall accuracy (0.9081), particularly when combined with the SMOTEENN rebalancing technique. Logistic Regression showed intermediate performance when used alongside SMOTETomek, with an F1-score of 0.2674, a ROC AUC of 0.7034, and an accuracy of 0.8478. By contrast, Support Vector Machines and K-Nearest Neighbors performed poorly in detecting referral cases, as reflected by F1-scores below 0.15 and ROC AUC values under 0.58, suggesting weak discriminative ability and limited applicability in this setting despite the application of resampling strategies.

The observed results indicate clear differences in how various machine learning techniques handle hospital referral prediction when applied to highly imbalanced clinical data. Conventional classifiers, including Logistic Regression and Random Forest, achieved reasonably high accuracy values (0.85 and 0.91, respectively); however, their corresponding F1-scores remained limited (0.27 and 0.31), highlighting persistent difficulties in identifying minority-class cases even when rebalancing methods such as SMOTETomek and SMOTEENN were applied. Support Vector Machines and K-Nearest Neighbors exhibited even weaker performance, with F1-scores below 0.15 and ROC AUC values under 0.58, suggesting poor generalization capacity in this challenging prediction task.

By comparison, the deep neural network showed a marked improvement, attaining an F1-score of 0.56 and a ROC AUC of 0.90 while preserving high overall accuracy (0.93). These results indicate that combining nonlinear deep learning models with dimensionality reduction and advanced resampling strategies is more effective at capturing the complex clinical relationships present in the data. Nevertheless, the moderate F1-scores observed across all evaluated approaches underline the intrinsic difficulty of forecasting infrequent referral events and point to the need for continued research focused on enhancing clinically meaningful precision and recall.

**Figure 4:**
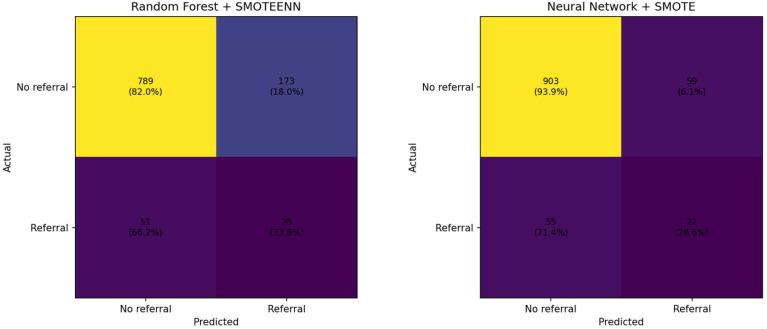
Confusion matrices for Random Forest + SMOTEENN and Neural Network + SMOTE models on the test set.

In order to better assess the clinical relevance of the predictive models under severe class imbalance, class-specific performance metrics for hospital referrals were additionally analyzed. As shown in Table 3 and Figure 4, the Random Forest model achieved a slightly higher recall for referral cases, but at the cost of a very low precision and a large number of false positives. In contrast, the Neural Network model exhibited a more conservative behavior, substantially improving precision and specificity while maintaining a comparable level of recall. This resulted in a higher F1-score and balanced accuracy for the referral class.

## 4. Discussion

The sustained increase in chronic diseases and the high fragility of the institutionalized elderly population pose a growing challenge for healthcare systems, both clinically and economically [49,50]. This situation leads to greater resource consumption and underscores the need to develop innovative care models that optimize efficiency, ensure quality of care, and reduce risks associated with unnecessary hospital transfers [51]. In this context, telemedicine emerges as a viable strategy, supported by emerging evidence suggesting benefits for both patients and healthcare systems in terms of safety, efficacy, and cost-effectiveness [31].

The most frequent diagnoses identified through telemedicine consultations include urinary tract infections, psychomotor agitation, hypertensive crises, metabolic disturbances, and falls, which align with the clinical profiles of institutionalized older adults described in previous studies. Both the nature of the diagnosis and the timeliness of telemedicine-supported assessments play a critical role in referral decisions. Acute conditions necessitating advanced diagnostic tools or invasive interventions, such as traumatic brain injury, severe respiratory compromise, or refractory metabolic decompensation, are more likely to warrant hospital referral. Conversely, conditions such as uncomplicated infections, medication-related issues, or behavioral symptoms are often effectively managed within the residential setting.

Our results demonstrate that the deep learning model implemented within the telemedicine system exhibits high operational efficiency and a presumptively favorable cost–benefit profile. The ability to assess patients’ conditions in real time via remote connection with residential care staff enables rapid clinical decision-making [52], reducing reliance on the physical presence of medical professionals and expanding the geographical coverage of the service [32]. This finding aligns with previous studies highlighting the utility of artificial intelligence-based systems to optimize care for vulnerable patients without replacing the critical role of healthcare personnel [33,34].

Contrary to the perception that deep learning systems might substitute medical staff, our results reinforce the concept that these tools serve as support for clinical decision-making. The integration of remotely obtained data with the patient’s complete medical history and comparable prior clinical events allows for robust comparative evaluation, facilitating a rapid and accurate response to acute events. This approach is consistent with the literature emphasizing the role of artificial intelligence in improving geriatric care, especially in settings with limited human resources [36,37,38], while also aligning with recent perspectives that stress the need for responsible deployment and regulatory compliance of AI-based clinical decision-support systems throughout their lifecycle, particularly when such tools are intended for use as certified medical devices in routine telemedicine practice [53].

Although global metrics such as AUC summarize overall discrimination, clinical deployment requires defining decision thresholds that balance sensitivity and specificity according to care priorities and resource availability. In telemedicine-supported acute care for nursing home residents, misclassification has direct clinical implications: false negatives may result in failure to escalate care when needed, leading to delayed diagnosis, clinical deterioration, and preventable complications in frail older adults, whereas false positives may prompt avoidable hospital transfers, increasing the risk of delirium, functional decline, and psychological distress, as well as placing additional strain on caregivers and healthcare resources. Consequently, threshold selection should be guided not only by statistical performance but also by a careful consideration of the clinical risks associated with both under- and over-referral in this vulnerable population.

While the proposed deep learning model did not substantially increase recall for hospital referral cases compared to the Random Forest baseline, it achieved markedly higher precision and specificity, thereby reducing false positive predictions. From a clinical perspective, this trade-off is highly relevant, as excessive false alarms may lead to unnecessary escalations and alert fatigue among healthcare professionals and patients. In this sense, improving the reliability of alerts may contribute to more appropriate clinical responses and avoid unnecessary care escalation.

Furthermore, the implementation of telemedicine is associated with a reduction in hospital admissions and transfers from residential care facilities, which has direct implications for patient experience and system sustainability [54,55]. The decrease in transfers helps to reduce episodes of disorientation, agitation, and the risk of pressure injuries, thereby improving resident well-being and preventing complications related to hospitalization [35]. From an economic perspective, this reduction in transfers and hospitalizations translates into significant healthcare resource savings by lowering both the direct costs of hospitalization and the indirect costs associated with transfer-related complications. These findings are consistent with previous studies reporting similar benefits in terms of safety and economic efficiency within the geriatric population [54,56].

The Comitas device has been instrumental in integrating telemedicine into our centers, enabling not only remote management of acute events but also comprehensive daily monitoring of residents. Its capabilities to measure vital signs, perform electrocardiograms, ultrasounds, and dermatological assessments, and conduct evaluations provide clinically relevant information that is incorporated into the patient’s medical record, thereby avoiding unnecessary referrals. Initial implementation required investment in equipment and specialized staff training, underscoring the importance of multidisciplinary education to ensure system safety and efficacy, a critical aspect to consider when adopting innovative technologies in complex clinical settings [57].

The limitations of this study must be carefully considered when interpreting the results. While the integration of telemedicine and deep learning models shows promising potential to enhance acute care management in nursing homes, challenges remain. These include the need for robust external validation, improved model interpretability, and comprehensive assessment of clinical outcomes beyond predictive performance metrics.

Although Truncated SVD improves computational tractability, the resulting latent components reduce direct feature interpretability, which is particularly relevant in clinical decision-support settings. Consequently, while the selected configuration optimizes predictive performance and scalability, future work will explore more interpretable dimensionality reduction strategies or post hoc explainability techniques to enhance clinical transparency.

Additionally, implementation barriers such as infrastructural demands and the necessity for extensive staff training must be addressed to enable wider adoption. Future research should focus on longitudinal studies that evaluate not only the predictive accuracy but also the real-world impact on patient health, healthcare utilization, and economic sustainability. Addressing these limitations will be essential to fully realize the transformative potential of telemedicine combined with artificial intelligence in improving geriatric care quality and healthcare system resilience.

## 5. Conclusions

Clinical results indicate that telemedicine enables numerous clinical situations to be treated on site, without the need to refer patients to the hospital. In most cases, these situations were resolved without external assistance by prescribing or modifying treatment, and in a significantly smaller number of cases, out-of-hospital emergency services had to be called upon to provide care in the residential setting. In this context, hospital referrals constituted a minority of cases, corresponding to patients necessitating escalation beyond the scope of telemedicine-facilitated management.

Informed by these clinically derived outcomes, this study evaluated multiple machine learning models to predict hospital referrals for acute cases in nursing homes, using a large and imbalanced dataset collected through a telemedicine platform. Our findings demonstrate that while traditional models provide moderate performance, the deep learning architecture leveraging advanced preprocessing and resampling methods offers a more balanced and reliable decision-support behavior by improving precision and specificity while maintaining comparable sensitivity. These results support the integration of AI-based decision support tools into telemedicine workflows to improve acute care management, taking into account two major situations to avoid: delays in necessary referrals and unnecessary referrals/transfers.

AI-assisted telemedicine represents a viable and effective strategy for managing acute events in institutionalized older adults, offering potential benefits for both patients and healthcare systems. However, several challenges remain. The moderate F1 scores observed reflect the inherent difficulty in predicting rare clinical events. Key limitations included the integration of heterogeneous data sources, and ensuring acceptance and adoption by healthcare personnel.

## Figures and Tables

**Table 1:** Hyperparameters and Training Configuration of the Final Deep Learning Model.

Category	Parameter	Setting
Input representation	Feature dimension	200 components after SVD
Model structure	Hidden layer sizes	256-128-64
	Activation function	LeakyReLU (α = 0.1)
	Output layer	Single neuron with sigmoid activation
Regularization	Dropout rates	0.3 (layers 1–2), 0.2 (layer 3)
Optimization	Optimizer	AdamW
	Learning rate	1 × 10^−3^
	Weight decay	1 × 10^−5^
	Loss function	Binary cross-entropy
Training procedure	Batch size	64
	Maximum epochs	150
	Validation proportion	10%
	Early stopping	Patience = 10 (validation loss)

**Table 2:** Experimental results.

Model	Accuracy	ROC AUC	F1-Score	Rebalancing Method
Logistic Regression	0.8478	0.7034	0.2674	SMOTETomek
Random forest	0.9081	0.7784	0.3148	SMOTEENN
SVM	0.7571	0.5739	0.1311	SMOTEENN
K-Nearest Neighbors	0.4413	0.5394	0.1400	SMOTETomek
Neural Network	0.9288	0.8971	0.5595	SMOTE

**Table 3:** Minority-class metrics on the test set for Neural Network model and Random Forest.

Model	Precision	Recall	F1-Score	Specificity
Random Forest (SMOTEENN)	0.131	0.338	0.188	0.820
Neural Network (SMOTE)	0.272	0.286	0.278	0.939

Performance Metrics are reported for the hospital referral class (minority class) and were computed on the independent test set.

## Data Availability

The datasets generated and/or analyzed during the present study are not publicly available due to privacy and ethical restrictions related to patient confidentiality. Further information may be available from the corresponding author upon reasonable request.
